# Supplementary material for: Exploring adolescent engagement in sexual and reproductive health research in Kenya, Rwanda, Tanzania, and Uganda: A scoping review
Source: PLOS Glob Public Health. 2022 Oct 19;2(10):e0000208. doi: 10.1371/journal.pgph.0000208 (PMC10022240; doi:10.1371/journal.pgph.0000208)
Supplement: S1 Text — A text file of the search strategy we used in this scoping review. (DOCX) [file pgph.0000208.s002.docx]

**S1 Text. Search Strategy**

Search terms were generated based on the research question and broken down into keywords, including synonyms and related terms. Keywords were combined using Boolean operators “OR” and “AND”. See below for a sample search strategy through OVID Medline:

(adolescen*.mp. OR teen*.mp. OR youth*.mp.) AND (Sexual Health OR Reproductive Health OR “sexual and reproductive health”.mp.) AND (East Africa.mp. OR Tanzania.mp. OR Rwanda.mp. OR Kenya.mp. OR Uganda.mp.)
