# Supplementary material for: Exploring adolescent engagement in sexual and reproductive health research in Kenya, Rwanda, Tanzania, and Uganda: A scoping review
Source: PLOS Glob Public Health. 2022 Oct 19;2(10):e0000208. doi: 10.1371/journal.pgph.0000208 (PMC10022240; doi:10.1371/journal.pgph.0000208)
Supplement: S1 Data — (DOCX) [file pgph.0000208.s003.docx]

**S1 Data**

| Author, title, year | Country of Study | Aims/objectives | Design/methods | Results/conclusions |
| --- | --- | --- | --- | --- |
| Adaji et al., (2010), The Attitudes of Kenyan In-School Adolescents Toward Sexual Autonomy | Kenya | Investigate attitudes of in-school adolescents in Kenya for insight to address SRH needs | Quantitative, Likert scale with secondary school students | Kenyan adolescents have conservative views towards premarital sex but may not translate into practice; strong influence of religion and gender norms |
| Atuyambe et al., (2015), Understanding sexual and reproductive health needs of adolescents: Evidence from a formative evaluation in Wakiso district, Uganda | Uganda | Assess the SRH needs of adolescents in Uganda and explore attitudes towards current services available | Qualitative, focus groups (FGs) with adolescents in and out of school | Four main themes: main adolescent health problems (HIV/STIs, unwanted pregnancy, gender dynamics), adolescent SRH needs (condoms, abortion care, adolescent specific care), health seeking behaviour and attitudes towards services (adolescents take little action when health needs come up but if problems persist they go to health facility) and preferred services and modalities for their provision |
| Bastien et al., (2009), Exposure to information and communication about HIV/AIDS and perceived credibility of information sources among young people in northern Tanzania | Tanzania | Explore exposure to HIV/AIDS info and communication among in and out of school adolescents in Tanzania | Quantitative, questionnaire (Likert) with adolescents | Young people in school reported more frequency of communication about HIV/AIDS information; preferred communicators of SRH information were friends, medial doctors |
| Cleeve et al., (2017), Abortion as agentive action: reproductive agency among young women seeking post-abortion care in Uganda | Uganda | Explores reproductive agency in relation to unsafe abortion among women seeking post-abortion care in Uganda | Qualitative, semi-structured interviews to gather perspectives of young women | Power imbalances/gender norms and contraception misconceptions (limited negotiating capacity among young women, gender-based violence, economic dependency); avoidance of stigma is a factor in accessing/not accessing care |
| Coast et al., (2019), Adolescent Sexual and Reproductive Health in Ethiopia and Rwanda: A Qualitative Exploration of the Role of Social Norms | Rwanda (and Ethiopia) | Examines how adolescent experience of SRH evolves during their adolescence | Qualitative, body mapping, community mapping, vignettes with adolescents and the greater community | Significant perceptions of SRH shaped in early adolescence (under 15); meeting SRH needs especially for adolescent girls is challenging because of misinformation; unsafe abortion is critical SRH issue |
| Damien et al., (2020), Youth underrepresentation as a barrier to sexual and reproductive healthcare access in Kasulu district, Tanzania: A qualitative thematic analysis | Tanzania | Explore how underrepresentation in decision making affects youth access to SRH services in rural community | Qualitative, DGs, interviews and observation at health facilities | Passive representation of youth in decision making, gender dynamics in who holds power (men), economic vulnerability of youth undermines influence on decisions and access to SRH services |
| Dangat & Njau, (2013), Knowledge, attitude and practices on family planning services among adolescents in secondary schools in Hai district, northern Tanzania | Tanzania | Determine knowledge, attitude and practices of secondary school students on family planning services in Tanzania | Quantitative, questionnaire | More than 2/3 of respondents had “adequate” knowledge of family planning services although use of services was low; family planning associated with infertility, reduction in sexual pleasure, increase in promiscuity |
| Godia et al., (2014), Young people’s perception of sexual and reproductive health services in Kenya | Kenya | Explore SRH problems young people face as well as their perceptions of available SRH services in Kenya | Qualitative, FGs, interviews | Problems adolescents face in regards to SRH include socioeconomic environment, STIs, lack of guidance on SRH, early and unprotected sex; addressing these needs includes more contraception, support from parents, understanding that girls are not solely responsible |
| Kemigisha et al., (2019), Evaluation of a school based comprehensive sexuality education program among very young adolescents in rural Uganda | Uganda | Evaluate the effectiveness of comprehensive SRH intervention in Uganda | Mixed methods, RCT and FGs to access SRH knowledge, sexual well-being and attitudes, sexual behaviour | Very young adolescents had improved SRH knowledge post-intervention; knowledge is not sufficient to drive behaviour change |
| Kemigisha et al., (2018), Sexual health of very young adolescents in South Western Uganda: a cross-sectional assessment of sexual knowledge and behaviour | Uganda | Investigate status of sexual behaviour, knowledge and information seeking behaviour among adolescents (aged 10-14) in Uganda | Quantitative, cross-sectional survey | Most respondents knew about HIV and puberty; 59% of respondents in study mentioned abstinence as contraceptive, 56% mentioned condoms; adolescents access information through radio; parents and schools should serve as valuable resources for adolescents in mitigating potential harms of misleading SRH info |
| Kiapi-Iwa & Hart, (2004), The sexual and reproductive health of young people in Adjumani district, Uganda: Qualitative study of the role of formal, informal and traditional health providers | Uganda | Explore what the best ways of improving young people’s access to sexual health care are in Uganda | Qualitative, in-depth interviews with young people and service providers | Adolescents had some knowledge of STDs but not fully informed; healthcare workers unsympathetic to needs of YP regarding contraception and pregnancy; stigma from healthcare workers is barrier to contraceptive use |
| Kipp et al., (2007), Adolescent reproductive health in Uganda: Issues related to access and quality of care | Uganda | Explore the important barriers, solutions identified by youth to reproductive care services in Uganda | Qualitative, in-depth interviews with key informants (adolescents not directly engaged) | Adolescents perceived as not experiencing reproductive health services as “user friendly”, lack of privacy and confidentiality, adolescents fear pregnancy, parents don’t talk about sex  “When asked about how adolescents perceived their risk of HIV infection and pregnancy, most health professionals felt that adolescents fear pregnancy much more than HIV infection.” (388-89) |
| Rehnström et al., (2019), Abortion and contraceptive use stigma: a cross-sectional study of attitudes and beliefs in secondary school students in western Kenya | Kenya | Measure stigmatizing attitudes and beliefs regarding abortion and contraceptive use among secondary school students in Kenya | Quantitative, questionnaires as part of RCT among secondary school students | Strongest theme explained that “a girl who has an abortion is committing a sin” and “a girl who uses contraception will encourage others to be promiscuous”; shame in the community and among individuals who have had an abortion; abortion and contraceptives considered harmful and leading to infertility |
| Madeni et al., (2011), Evaluation of a reproductive health awareness program for adolescence in urban Tanzania-A quasi-experimental pre-test post-test research | Tanzania | Evaluate reproductive health awareness program for improvement of SRH for unmarried adolescent girls/boys in urban Tanzania | Quantitative, questionnaire (Likert) | School-based programs were effective for knowledge improvement but attitude difficult to change |
| Maly et al., (2017), Perceptions of Adolescent Pregnancy Among Teenage Girls in Rakai, Uganda | Uganda | Explore perceptions of adolescent pregnancy among currently pregnant and sexually active teenage girls in Rakai district Uganda | Qualitative, in-depth interviews | Perceptions of pregnancy (i.e. readiness and control over getting pregnant); perceptions impacted by structural factors such as cultural norms, stigma; agency among adolescent girls limited due to gender norms, economic vulnerability |
| Mason et al., (2013), “We keep it secret so no one should know” - A qualitative study to explore young schoolgirls attitudes and experiences with menstruation in rural Western Kenya | Kenya | Examine girls’ attitude, experiences and concerns around menstruation | Qualitative, FGs | Limited knowledge on menarche; menstruation associated with maturity; shame and fear exists around menarche |
| Michielsen et al., (2014), Rwandan young people’s perceptions on sexuality and relationships: Results from a qualitative study using the ‘mailbox technique.’ | Rwanda | Gain understanding of young Rwandan’s perceptions on sex and relationships to formulate effective SRH promotion interventions | Qualitative, “mailbox technique” | Young people are curious and experiment with sex; limited capacity of young people to deal with sexual vulnerability (unprepared because limited information about bodies); abstinence a key theme |
| Mitchell et al., (2006), Social scripts and stark realities: Kenyan adolescents’ abortion discourse | Kenya | Explore Nairobi young people’s scripts on unintended pregnancies and abortion decision-making | Qualitative, arts-based/narrative | Abortion was discouraged among participants and marriage was seen as solution; framing of views on SRH around religion; students seemed to have one set of personal behaviours advocated in abstract and another enacted in the face of actual events; gap in what is learned at school and what is practiced |
| Mkumbo, (2010), What Tanzanian young people want to know about sexual health; implications for school-based sex and relationships education | Tanzania | Assess adolescent views about provision of sex and relationship education in Tanzania and investigate what adolescents would like to learn about SRH | Qualitative, questionnaire among adolescents | Misconceptions on condom use prevalent (i.e. some students asked if it is good to use condom with loving partner); misconceptions about STIs/HIV; adolescent needs and preferences for sex education are more diverse than believed (i.e. want to know about pleasure, masturbation not just HIV/STIs) |
| Mtasingwa, (2020), Challenges Facing Tanzanian Youth in the Fight Against HIV/AIDS: Lessons Learnt from Mbeya Region, Southern Highlands | Tanzania | Provide understanding of challenges youth face in Mbeya region in regards to HIV mitigation | Qualitative, FGs, in-depth interviews | All youth involved were aware of HIV, received information from radio, TV and peers; more than ¼ of respondents indicated they did not have sufficient SRH information; suspicion of condoms because they are free of charge; significant influence of religious/parental beliefs that construct SRH as immoral; lack of parents as role models; need to engage youth more in SRH programming |
| Muhwezi et al., (2015), Perceptions and experiences of adolescents, parents and school administrators regarding adolescent-parent communication on sexual and reproductive health issues in urban and rural Uganda | Uganda | Explored parent-child communication on SRH | Qualitative, FGs | Mothers more comfortable talking with adolescents about SRH; adolescents get SRH information from peers; most SRH communication is about STIs/HIV |
| Mung’ong’o et al., (2010), Knowledge, Attitude and Practice on Contraceptive Use among Secondary School Students in Dar es Salaam, Tanzania | Tanzania | Determine contraceptive prevalence of use, knowledge and attitude among secondary school students in Dar es Salaam Tanzania | Quantitative, questionnaire to measure contraceptive use | 73% of participants had good to average knowledge on contraceptives, most were over age 16; most participants had good to average knowledge of contraceptives but that did not translate to increased level of use |
| Mutea et al., (2020), Access to information and use of adolescent sexual reproductive health services: Qualitative exploration of barriers and facilitators in Kisumu and Kakamega, Kenya | Kenya | Describe barriers to and facilitators of access to adolescent SRH services in Kisumu and Kakamega counties in Kenya | Qualitative, in-depth interviews, FGs engaging adolescents, healthcare workers, teachers, community leaders | Lack of access to contraceptive services and methods a challenge; economic barriers to accessing services; SRH not discussed at home due to discomfort with topic; confidentiality concerns and religious influence a barrier at community level |
| Nalwadda et al., (2010), Persistent high fertility in Uganda: Young people recount obstacles and enabling factors to use of contraceptives | Uganda | Explored views about obstacles and enabling factors for contraceptive use among young people aged 15-24 in Uganda | Qualitative, FGs | Obstacles to contraceptive use included misconceptions and fears (i.e. contraceptives interfere with fertility, condoms dangerous to uterus); fear of parents reaction to contraceptive use; gender power relations impact women’s ability to decide; sociocultural norms impact use of contraceptive (stigma) |
| Nkurunziza et al., (2020), Breaking barriers in the prevention of adolescent pregnancies for in-school children in Kirehe district (Rwanda): A mixed-method study for the development of a peer education program on sexual and reproductive health | Rwanda | Contribute to reduction of adolescent pregnancies by obtaining empowered adolescents who make more and better use of means to prevent adolescent pregnancy | Mixed methods, questionnaire and FGs | No results presented |
| Nyblade et al., (2017), Perceived, anticipated and experienced stigma: exploring manifestations and implications for young people’s sexual and reproductive health and access to care in North-Western Tanzania | Tanzania | Explore micro-level stigmatization process by analyzing presence, forms and potential implications for adolescent access to SRH information and services in Mwanza | Qualitative, FGs and in-depth interviews | Stigma reported by respondents around SRH – negative name calling re sexual deviance among young women, shaming by family due to sexual activity, social and physical isolation/shunning from community, fear of being punished for engaging in sexual activity; adolescents avoid accessing contraceptives at health facilities due to fear of lack of confidentiality, stigma, negative labelling |
| Palomino González et al., (2019), The knowledge-risk-behaviour continuum among young Ugandans: What it tells us about SRH/HIV integration | Uganda | Establish knowledge levels, attitudes and practices related to SRH among adolescents in Uganda | Quantitative, survey | Knowledge levels of STIs, family planning and HIV across all young people was high – increasing HIV prevalence and high rates of teenage pregnancy in Uganda are not because of lack of information/knowledge; risky sexual behaviour despite level of knowledge |
| Plummer et al., (2008), Aborting and suspending pregnancy in Rural Tanzania: An ethnography of young people’s beliefs and practices | Tanzania | Explores experiences of rural girls and young women who attempt abortions but have complications (or not) which they do not seek care for at hospital | Qualitative, observation, semi-structured in-depth interviews | Adolescents conceal sexual relationships because social norms, condoms perceived as negative; abortion is stigmatized but widespread (although infrequent) |
| Remes et al., (2010), Dusty discos and dangerous desires: Community perceptions of adolescent sexual and reproductive health risks and vulnerability and the potential role of parents in rural Mwanza, Tanzania | Tanzania | Explore perceptions of SRH and HIV risk-reduction interventions | Qualitative, questionnaires, face to face surveys, interviews, FGs | Participants linked SRH vulnerability to adolescent aspirations to lead a “modern life” (i.e. fashionable clothes mentioned as causative factor of early sexual relationships and transactional sex); parent/child communication about SRH limited; Adolescents felt increased SRH risk behaviour was caused in part by lack of responsibility of parents and caregivers not providing adequate support, food, shelter, role model etc.; greater awareness of SRH does not translate to behaviour change; distrust of condoms |
| Sebti et al., (2019), Child and youth participation in sexual health-related discussions, decisions, and actions in Njombe, Tanzania: A focused ethnography | Tanzania | Explore how children/youth in Njombe participate in discussions, decisions and actions regarding their sexual health | Participatory, ethnography, group interviews, observation | Knowledge and understanding of sexual health – sexual health defined as being free from STIs, no infections, “knowing your status”; sources of information about SRH include radio, TV, school; knowledge on SRH does not correlate to understanding SRH; adolescents value learning from adults, peers about SRH |
| Sommer, (2009), Ideologies of sexuality, menstruation and risk: Girls’ experiences of puberty and schooling in northern Tanzania | Tanzania | Capture accounts of girls in their own bodies during transition in puberty – intersection of menses, puberty and schooling | Participatory, observation, document analysis, in-depth interviews with girls and adults | Fear, shame and confusion around menses – breakdown of traditional communication about menses in urban areas because of schools, limited communication with parents/aunts; confusion around what menarche means in terms of sex/virginity |
| Stats et al., (2020), Knowledge and misconceptions surrounding family planning among Young Maasai women in Kenya | Kenya | Explore knowledge and perceptions of family planning methods among young women in Laikipia County, Central Kenya | Qualitative, in-depth interviews semi-structured questionnaire with Maasai women | Women were uncertain of what family planning was or assumed family planning was only an injection; idea that family planning was only reserved for women who had already had children; perceptions of family planning associated with infertility |
| Tavrow et al., (2012), Community norms about youth condom use in Western Kenya: is transition occurring? | Kenya | Analyzed qualitative data to determine how different categories of adults and students in Kenya view adolescent condom use | Qualitative, FGs with adolescents | 50% of participants thought youths who have condoms inherently engage in unacceptable or deviant behaviour; condom use viewed as deviant; stigma around condom use; girls saw condom use among boys as a way to dominate sexual decision making |
| Wolf et al., (2015), The effectiveness of an adolescent reproductive health education intervention in Uganda | Uganda | Examine adolescents’ baseline STI and contraceptive knowledge, determine if knowledge varies by demographic factors, evaluate program | Quantitative, surveys | Adolescents had lower baseline knowledge of contraceptives compared to STIs |
