# Supplementary material for: Exploring adolescent engagement in sexual and reproductive health research in Kenya, Rwanda, Tanzania, and Uganda: A scoping review
Source: PLOS Glob Public Health. 2022 Oct 19;2(10):e0000208. doi: 10.1371/journal.pgph.0000208 (PMC10022240; doi:10.1371/journal.pgph.0000208)
Supplement: Attachment — Submitted filename: Response to Reviewers.docx [file pgph.0000208.s004.docx]

Dear Dr. Seth Christopher Yaw Appiah & Reviewers,

First, we would like to thank you and each of the reviewers for your insightful comments on our submitted manuscript. They have been really helpful in refining and clarifying the message of the manuscript.

Please find our response to the comments explained in this letter and indicated specifically in the ‘Revised Manuscript with Track Changes’.

| **Reviewer/Editor Comment** | **Authors’ Response** |
| --- | --- |
| **Handling Editor** | |
| Please provide separate figure files in .tif or .eps format only and remove any figures embedded in your manuscript file. | Figure 1 – PRISMA Flow Diagram has been resubmitted in .tif format. |
| Please note that your Data Availability Statement is currently missing the repository name and/or the DOI/accession number of each dataset OR a direct link to access each database. | All data are available within the manuscript, references and Supporting information; therefore, the Data Availability Statement can be amended to, “All relevant data are within the manuscript, references and Supporting information files.” |
| Please review your reference list to ensure that it is complete and correct. | The reference list has been reviewed and corrections have been made, including the correct doi format. |
| The authors have provided an important topic of contemporary relevance to the world and particularity the East African region with relatively very youthful population. The question on why some East African Countries were excluded have not justifiably been explained. Authors will do a great benefit to readers by providing justification for or upgrading the scope to reflect the entire East African Countries. In going through appendix 2, it appear the authors in using the Boolen operators did not include countries such as Burundi, South Sudan, Ethiopia, Somalia and a few other East African Countries. This can be confirmed in lines 70-74 of the manuscript. While appendix 3 presents the summary of the findings, it will be better a section is created in the tale to indicate where each of the study is being conducted. A major limitation of this paper is the absence of 'where the study was conducted' as a central criteria informing the data extraction framework. The use of the lead authors affiliation may not suffice and may potentially lead to exclusion of several significant articles addressing the topic if the study setting/location is not included as a central criteria . These issues ought to be addressed significantly. The authors could equally re-align their title to reflect ' in selected countries in Africa' to reflect the accurate submission of the paper. | Thank you, this is a great point and helpful in refining the language of the manuscript further. The primary author is a PhD student and as part of her dissertation had been working with organizations in East African Community (EAC) member countries. Through discussions with in-country organizations and consideration of OECD data, we decided the selected four countries were a good frame of focus to inform the next phases of the research. We have adjusted the language of the manuscript to reflect the intended, more precise meaning of including Kenya, Rwanda, Tanzania, and Uganda. We have also added justification for the focus on pp. 4, lines 63-69.  Appendix 3 has also been updated to reflect the country of focus in each study to clarify the inclusion of studies based on country, not lead author affiliation institution. In addition, we have shifted the language in the Methods section on lines 91-108 to clarify the inclusion/exclusion criteria and how studies were selected, to further highlight that a central criteria of selecting studies was based on “where the study was conducted”. We have also added the results of studies included, based on specific country location, on lines 131-133.  The title has also been realigned to reflect the precise countries of focus. |
| **Reviewer #1** | |
| The review is very descriptive. I miss a clear theoretical frame that guides the review process. In the results section I am left wondering the so what question. What can we do with this information. What policies and programs can benefit from this evidence? The scope is too broad hence the novelty of the evidence is thin. | We appreciate this feedback and have reviewed the PLOS Criteria for Publication. Aligned with the criteria, this manuscript presents original research with conclusions supported by the data consolidated.  We confirm the methodology from Arksey and O’Malley for Scoping Reviews which supports the validity and rigour of our approach to thematically present new ideas and ways forward, emphasizing the importance of engaging adolescents in ASRH research. The findings presented in our manuscript will inform context specific ASRH policies and programs with an emphasis on prioritizing adolescents in the development of such policies and programs.  We appreciate the Handling Editor’s remarks acknowledging that the paper will not be rejected on the grounds of inadequate novelty. |
| **Reviewer #2** | |
| The abbreviated term ASRH is in the abstract but is not mentioned in the introduction, I consider the authors should place the term here too before abbreviating it. | The full term is included on line 33 in the introduction, before abbreviating to ASRH. |
| The objective of the research is clear. However, it would be helpful if it was specified in the title and the abstract that what the authors are trying to find through the review is how adolescents are engaged on ASRH research because at first sight, it just seems that they are engaged WITH ASRH, instead of the research specifically. | Title and abstract have been refined to better articulate the aim of the review on how adolescents are engaged in ASRH research. |
| I consider it would be important if the authors could briefly explain why they chose East Africa as the research region. This could give the manuscript a little bit more context for the reader. | This is a helpful point – thank you. Justification on why the focus of the article is on East Africa in general is explained on lines 41-55, where a gap in knowledge exists on how adolescents are engaged in ASRH research.  As mentioned in the comments to the Handling Editor, the primary author is a PhD student and as part of her dissertation had been working with organizations in East African Community (EAC) member countries. Through discussions with in-country organizations and consideration of OECD data, we decided the selected four countries were a good frame of focus to inform the next phases of the research. We have adjusted the language of the manuscript to reflect the intended, more precise meaning of including Kenya, Rwanda, Tanzania, and Uganda. We have also added justification for the focus on pp. 4, lines 63-69. |
| It caught my attention that “funding” was included in the Data extraction framework (Table 1), so I think if the authors will include it here, they could explain why this is part of the inclusion/exclusion criteria or why is it important for the review. | Funding was included in the data extraction framework as it was a relevant point of data collection at the start of the review. As themes emerged, the authors determined a discussion of funding to be out of scope. We have removed “Funder(s)” from Table 1. |
| In page 5 of the manuscript, I do not consider it is necessary to include the reviewers’ initials, they could be eliminated unless the authors want the readers to know who contributed in what section of the review. | Initials of the authors have been omitted. Further details on the role of each author are included in the limitations paragraph on lines 282-283. |
| When mentioning which articles apply to which statement (e.g. content of perspectives section), I consider it would be more helpful to place them as footnotes so that the reader can later go to the references section to find them. | We have not included footnotes as footnotes are not permitted based on journal requirements. |
| On page 8, the statement “The fear of infertility is connected to the high value placed on having children in East Africa” could use an explanation or small discussion on the reason the authors think or found out in this review why this is important. | Thank you, this is an important point. We have added the following discussion at lines 172-174 explaining why this statement is important: “Literature noted that adolescents are constrained by cultural and gender norms which equate having children with status and value, creating pressure to prove their fertility, especially among young women [43].” |
| I noted the references are not in alphabetical order, make sure they are before submitting the final version, thanks! | References are included according to “Vancouver” style as specified by PLOS, indicating that references are numbered in the order that they appear in text rather than alphabetically. |
| To make the paper more understandable, especially the methods section, establishing a little bit more clearly the inclusion/exclusion and how the studies were selected (although it is clear the main author and reviewers worked as a group) would be of great help. | We have shifted the language in the Methods section on lines 91-108 to clarify the inclusion/exclusion criteria and how studies were selected. |
| **Reviewer #3** | |
| Is there a compelling reason why only 4 East African countries were included in the scoping review? | This is a helpful point – thank you. As discussed above, we selected the four countries included in this study (Rwanda, Tanzania, Kenya, and Uganda) based on discussions with in-country organizations and consideration of OECD data. We have added this justification at lines 63-69. |
| The depiction of what constitutes ‘contents of perspectives for SRH’ is quite categorical. Any reference for this or is it an arbitrary determination? | We used this subheading, ‘Content of perspectives’, to capture the four themes emerging from the data including knowledge, sources of information, gaps in information, and adolescent friendly services. This subheading is not based on a reference or existing definition. |
| Was there a consideration of educational level as a possible determinant of participation by adolescents by any of the studies reviewed? | We have gone back and reviewed the studies, and have determined that educational level was not a determinant of adolescent participation in the research reported. Therefore, we have not included education level as a consideration in our analysis. |
| Was substance abuse appraised as a factor by any of the studies reviewed? | We have reviewed the studies and determined that substance abuse was mentioned as an issue that adolescents face in three articles and has been included in the results section at lines 195-197. |
| Aside the stated role of the primary author in sole extraction and analysis of data, the role of the other authors is somewhat ambiguous. Some clarification needed. | The role of each author has been further clarified on lines 281-283 as follows: “Although we recognize that best practice involves multiple reviewers, the input from contributing authors on conceptualization (LB, HC, PO, DD), data collection (PO, HC), and drafting and editing (HC, DD, AB) added to the rigour of ideas shared.” |

We thank each reviewer again for their thoughtful commentary. Please let us know if there is anything else we can further clarify.

On behalf of the team,

Hanna Chidwick
